# Stereotyped Combination of Hearing and Wind/Gravity-Sensing Neurons in the Johnston’s Organ of *Drosophila*

**DOI:** 10.3389/fphys.2019.01552

**Published:** 2020-01-08

**Authors:** Yuki Ishikawa, Mao Fujiwara, Junlin Wong, Akari Ura, Azusa Kamikouchi

**Affiliations:** Division of Biological Science, Graduate School of Science, Nagoya University, Nagoya, Japan

**Keywords:** ear, mechanosensory neuron, fruit fly, scolopidium, compartment

## Abstract

The antennal ear of the fruit fly, called the Johnston’s organ (JO), detects a wide variety of mechanosensory stimuli, including sound, wind, and gravity. Like many sensory cells in insect, JO neurons are compartmentalized in a sensory unit (i.e., scolopidium). To understand how different subgroups of JO neurons are organized in each scolopidial compartment, we visualized individual JO neurons by labeling various subgroups of JO neurons in different combinations. We found that vibration-sensitive (or deflection-sensitive) neurons rarely grouped together in a single scolopidial compartment. This finding suggests that JO neurons are grouped in stereotypical combinations each with a distinct response property in a scolopidium.

## Introduction

The ability to sense our surrounding environment is crucial for survival. Animals have developed specialized sensory organs to detect various external and internal signals. The ear is an example of a specialized organ that detects sound; in mammals, the ears are also responsible for balance. Some insect species also have a specialized hearing organ, the insect ear, which is located on different body parts, including the head, wings, thorax, abdomen, and legs (Gopfert and Hennig, 2016). Studies of the insect hearing system have yielded important findings ranging from the biophysics of sound perception to auditory signal processing in the brain (Coen and Murthy, 2016; Gopfert and Hennig, 2016).

**Figure 1:**
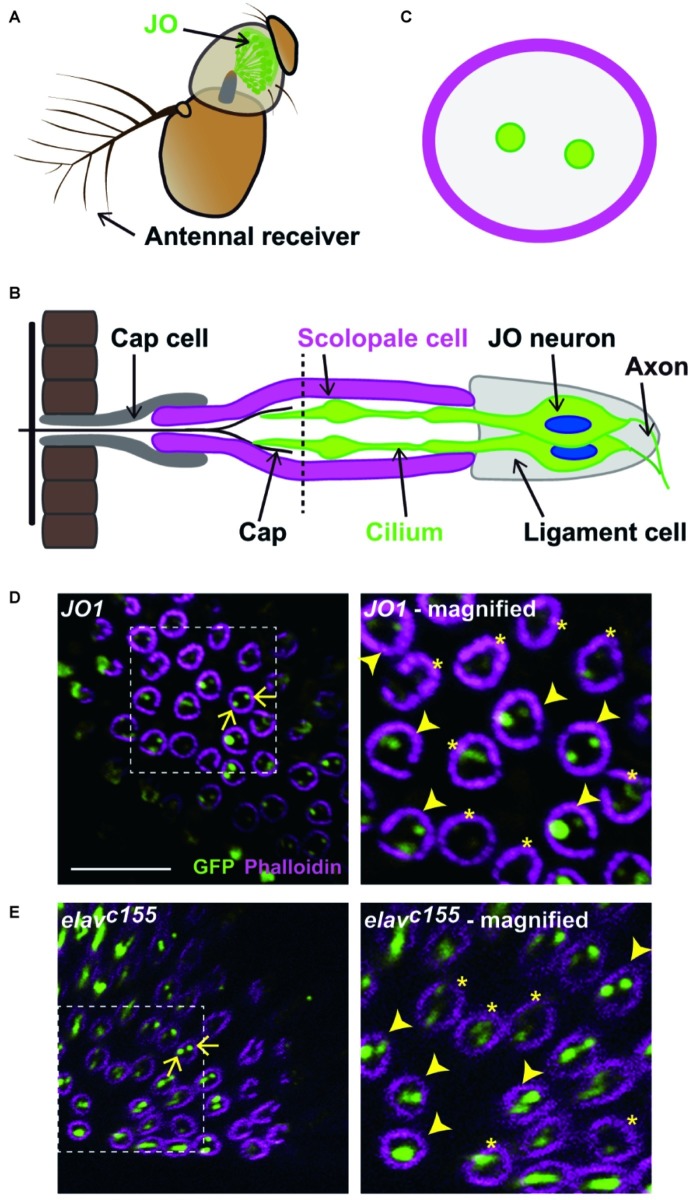
Labeled JO neurons in a scolopidium. **(A)** The antennal ear of fruit flies. Johnston’s organ (JO) is housed in the second antennal segment. **(B)** Scolopidium in the JO. A schema of a section at the dashed horizontal line is shown in **(C)**. **(C)** Horizontal view of a scolopidium. Labeled cilia are visualized as dots (green) in a scolopidium (magenta). **(D,E)** Labeled JO neurons in *GAL4* driver strains that label all JO subgroup neurons. Scolopidia containing *JO1-* **(D)** and *elav^c155^*-labeled cilia **(E)**. Arrows indicate the examples of labeled cilia (green) in a scolopidium (magenta) (left panels). Dotted square indicates the area that is magnified in the right panel. Arrowheads and asterisks in the magnified views indicate the selected and not-selected scolopidia, respectively (right panels). Scale bar = 10 μm. Panels **(A)** and **(B)** were modified from Ishikawa and Kamikouchi (2016) and Matsuo et al. (2016) with permissions.

The fruit fly *Drosophila melanogaster* is a useful model for studying the cellular and circuit mechanisms of hearing and other types of mechanosensory processing (Patella and Wilson, 2018). In fruit flies, the ear is comprised of the antennal receiver and hearing organ called the Johnston’s organ (JO) (Figure 1A). The JO, which is located at the second antennal segment, is the largest mechanosensory organ in fruit flies. Stimuli from an external source, such as sound, wind, or gravity, induce the movement of the antennal receiver, which activates the mechanosensory neurons in the JO, the JO neurons. JO neurons serve a vital role in the fruit fly behavior, such as the locomotor change in response to courtship sound, wind-induced suppression of locomotion, anti-geotaxis behavior, and flight control (Kamikouchi et al., 2009; Yorozu et al., 2009; Mamiya and Dickinson, 2015).

The fruit fly’s JO neurons are classified into five subgroups, JO-A to JO-E, according to their distinct projection patterns in the brain (Kamikouchi et al., 2006). These subgroups can further be categorized into vibration-sensitive and deflection-sensitive neurons (Kamikouchi et al., 2009; Yorozu et al., 2009). JO-A and JO-B neurons are vibration-sensitive, and function as sound sensors. Their response is frequency-dependent, ranging from ~10 Hz up to ~1,000 Hz when measured together. When measured separately, the JO-B neurons show a low frequency preference at about <100 Hz, while JO-A neurons preferentially respond to higher frequency (Matsuo et al., 2014; Patella and Wilson, 2018). On the other hand, JO-C and JO-E are activated maximally by static deflection of the antennal receiver, and serve as the major gravity and wind sensors. The precise function of JO-D neurons has not yet been determined due to their small total percentage (<30 neurons) and the lack of specific driver strains. Both vibration and deflection stimuli activate JO-D neurons (Matsuo et al., 2014).

An interesting feature of JO neurons is their compartmentalization; that is, two or three JO neurons are grouped together in specialized structures known as scolopidia (Todi et al., 2004). Each JO scolopidium acts as a sensory unit that helps to connect the apical sensory cilium to the joint cuticle. The scolopale cell wraps around the cilia of JO neurons and provides a sealed environment for maintaining and regulating the ionic composition of the endolymph (Figure 1B; Boekhoff-Falk and Eberl, 2014). Although previous findings suggest that JO neurons belonging to the same scolopidium should belong to heterogeneous subgroups (Kamikouchi et al., 2006), it remains unclear how different subgroups of JO neurons are organized in each scolopidium.

Similar examples of compartmentalized sensory neurons can be found in the sensilla that house the olfactory receptor neurons (ORNs) of *Drosophila*, the CO_2_-senstitive ORN of the malaria mosquito *Anopheles*, and the taste sensilla of flies and other insects, where the primary sensory neurons are grouped in stereotyped combinations (de Bruyne et al., 2001; Lu et al., 2007; Freeman and Dahanukar, 2015). In insect olfactory sensilla, it is predicted that short-term activation of one ORN may interfere with the signaling of a neighboring ORN (Nikonov and Leal, 2002; Vermeulen and Rospars, 2004), and in the case of *Drosophila*, transient excitation of one ORN inhibits the prolonged activation of the neighboring ORN (Su et al., 2012). This interaction between compartmentalized ORNs does not require a synaptic interaction, and might reflect ephaptic coupling.

To investigate how the different subgroups of JO neurons are organized in each scolopidium, we examined the distribution of JO neurons housed within the scolopidia. Our findings suggest that vibration- and deflection-sensitive JO neurons are housed as a pair in each scolopidium of the JO.

## Materials and Methods

### Fly Stocks and Culture

Flies were reared on yeast/cornmeal/agar medium at 25^°^C. The *GAL4* strains that label JO neurons (Kamikouchi et al., 2006; Ishikawa et al., 2017) are shown in Table 1. *elav^C155^* was used as a pan-neuronal *GAL4* driver (RRID: BDSC_458, obtained from the Bloomington *Drosophila* Stock Center). *UAS-nompC-L-GFP8* (Cheng et al., 2010), a gift from Dr. Y. N. Jan, was used as a reporter strain to express NompC-L-GFP.

**Table 1:** Labeling patterns of *GAL4* driver strains and the number of labeled JO neurons in each scolopidium.

*Group*	*GAL4* strains	Labeled subgroups	Observed antennas	Countable scolopidia	Selected scolopidia	Number of selected scolopidia		
						1 JO neuron	2 JO neurons	3 JO neurons
	*JO1*	A, B, C, D, E	3	99	65	1 (1.5%)	64 (98.5%)	0 (0%)
	*elav^C155^*	A, B, C, D, E	11	123	69	3 (4.35%)	65 (94.2%)	1 (1.45%)
*1*	*JO15*	A, B (most)	4	161	77	75 (97.4%)	2 (2.6%)	0 (0%)
	*JO31*	C, E (most)	3	117	54	54 (100%)	0 (0%)	0 (0%)
	*JO32*	C, E (most)	3	176	90	87 (96.7%)	3 (3.3%)	0 (0%)
	*JO15 + JO31*	A, B, C, E	8	229	88	64 (72.7%)	21 (23.9%)	3 (3.4%)
*2*	*R74C10*	A (most)	26	468	108	91 (84.3%)	16 (14.8%)	1 (0.9%)
	*JO22*	A (a few)	3	117	4	4 (100%)	0 (0%)	0 (0%)
	*JO24*	A (a few)	4	226	28	28 (100%)	0 (0%)	0 (0%)
	*JO26*	A (a few)	5	278	47	47 (100%)	0 (0%)	0 (0%)
	*JO2*	B (most)	7	206	77	77 (100%)	0 (0%)	0 (0%)
	*R74C10 + JO2*	A, B	5	290	107	101 (94.4%)	6 (5.6%)	0 (0%)

*Group 1 and group 2 are *GAL4* driver strains that label multiple and single subgroups of JO neurons, respectively. Group-1 *GAL4* driver strains label most JO-A and JO-B (*JO15*) or JO-C and JO-E (*JO31* and *JO32*) (Kamikouchi et al., 2006). In group 2, *R74C10* and *JO2* label most JO-A and JO-B neurons, respectively (Kamikouchi et al., 2006; Ishikawa et al., 2017). *JO22*, *JO24*, and *JO26* label a few JO-A neurons (Ishikawa et al., 2017)*.

### Immunohistochemistry

Female flies at 7–14 days after eclosion were used for dissection. Immunolabeling was performed as described previously with minor modifications (Matsuo et al., 2014). In brief, antennae were fixed with 4% paraformaldehyde in phosphate-buffered saline for 60–90 min on ice and labeled with antibody. As primary antibodies, we used rabbit green fluorescent protein (GFP) polyclonal antibody (Invitrogen, La Jolla, CA, #A11122; RRID: AB_221569; 1:1,000 dilution) to detect NompC-L-GFP and rat Elav antibody (Hybridoma Bank, Rat-Elav-7E8A10 anti-elav; 1:250 dilution) to detect JO neuron cell bodies. As secondary antibodies, we used Alexa Fluor 488-conjugated anti-rat IgG (Jackson ImmunoResearch, Cambridgeshire, UK, #112-545-167; RRID: AB_2338362; 1:300 dilution) and Alexa Fluor 647-conjugated anti-rabbit IgG (Thermo Fisher Scientific, #A21245; RRID: AB_2535813; 1:300 dilution). Alexa Fluor 555 phalloidin (Thermo Fisher Scientific, # A34055; 1:50 dilution) was used to visualize F-actin.

### Visualization of Antennal Morphology

For observation of the scolopidia from various angles, each of the second antennal segments was mounted on a slide glass at a different orientation except for the ventral and backward sides up; observation from these sides gives obscure images and thus we avoided these angles. Each sample was viewed under a confocal laser scanning microscope (FLUOVIEW FV1000D, Olympus, Japan) equipped with a silicone-oil immersion 60x Plan-Apochromat objective lens (NA = 1.30, UPLSAPO 60xS, Olympus). Each sample was scanned twice; the first series was to observe the overall structure and to locate the scolopidia and JO neurons with a resolution of 512 × 512 pixels (0.295 μm/pixel), and the second series was to focus on specific clusters of scolopidia and JO neurons with a resolution of 640 × 640 pixels (0.110 μm/pixel).

### Quantification of the Number of Labeled Johnston’s Organ Neurons in Scolopidia

The number of labeled JO neurons in each scolopidium was quantified by analyzing the confocal data series. Because the scolopidia are arranged in a radial fashion in the second antennal segment, each individual scolopidium is oriented at a different angle in a single image (Kamikouchi et al., 2006). The scolopidia that are mounted perpendicular to the cover glass appear as a circle while the others appear as ovals or rods when observed in the confocal sections. The cilia of JO neurons within the scolopidia with a circular appearance are visualized as dots when labeled and can be accurately quantified by counting the number of dots in each circle (Figure 1C). In contrast, quantification of the number of labeled JO neurons in the scolopidia appearing as a rod shape is unreliable due to the overlapping of multiple JO neurons and scolopidia in the same spot. To accurately count the number of labeled JO neurons in each scolopidium, only the scolopidia mounted perpendicular to the cover glass, thus appearing as a circle, were quantified in this study. At a certain confocal section, we sometimes observed only one labeled JO neuron, even the scolopidium actually housed two labeled JO neurons, which could be observed at a different section. This could be due to termination of the cilia of two JO neurons in each scolopidium at different levels along the longitudinal axis of the scolopidium (Uga and Kuwabara, 1965). To avoid underestimating the number of labeled JO neurons in each scolopidium due to the different cilia lengths, we scanned the scolopidium along its longitudinal axis so that cilia of all the labeled JO neurons could be observed.

Statistical analyses were performed using R (version 3.3.3). The ratio of single/double/triple-labeled neuron populations was compared by Fisher’s exact test. Values of *p* were adjusted using the Bonferroni method for multiple comparisons.

## Results

### Visualization of Labeled Cilia in Johnston’s Organ Scolopidia

In the JO of *Drosophila*, ~10% of the scolopidia contain three JO neurons scattered throughout the JO, whereas the remaining scolopidia contain two JO neurons (Todi et al., 2004). To clarify how different subgroups of JO neurons are distributed within each scolopidium, we visualized the cilia of specific JO neurons using the *GAL4/UAS* system to express NompC-L-GFP reporters (Cheng et al., 2010). First, to evaluate whether the NompC-L-GFP marker dependably visualizes the cilia of labeled JO neurons, we used two *GAL4* strains that label most JO neurons: *JO1* (a.k.a. *NP0761*), which labels 94% of JO neurons (Kamikouchi et al., 2006), and *elav^C155^*, which is widely used as a pan-neuronal *GAL4* driver.

To count the number of labeled cilia in each scolopidium, we used the scolopidia that appeared perpendicular to the confocal scanning plane for the analysis (Figure 1D). At this angle, each scolopidium was visualized as a circle and the cilia of JO neurons within it appeared as dots, which were easy to quantify (see section “Materials and Methods” for details). First, we screened the countable scolopidia visualized as circles. Next, we selected scolopidia in which at least one labeled cilium was clearly visualized among the countable scolopidia to avoid underestimating the proportion of labeled JO neurons by including the inappropriate angles or poor antibody permeable samples. Finally, we counted the number of labeled cilia and obtained the percentages of one, two, and three labeled JO neurons among the selected scolopidia.

We evaluated ~100 countable scolopidia in each of two *GAL4* strains. Approximately half of them exhibited strong and clear signals of one or more labeled cilia and were thus regarded as selected scolopidia, whereas the other half exhibited obscure signals, which were excluded from the following quantification (Figures 1D,E). More than 90% of the selected scolopidia had two labeled JO neurons in both strains (Figures 1D,E; Table 1). A few scolopidia (< 5%) had only one labeled JO neuron in both *GAL4* strains and scolopidia that had three labeled JO neurons were observed only when *elav^C155^* was used. On the basis of these findings, we concluded that the NompC-L-GFP reporter system was useful for evaluating the ratio of one, two, and three labeled JO neurons in a single scolopidium, although not all scolopidia could be evaluated.

### Organization of Vibration-Sensitive and Deflection-Sensitive Johnston’s Organ Neurons

To reveal the combination of JO neuronal subgroups in each scolopidium, we used other *GAL4* strains, each of which labels subsets of JO neuronal subgroups (Table 1; Kamikouchi et al., 2006; Ishikawa et al., 2017). We divided eight *GAL4* strains into two groups according to the combination of labeled subgroups (Table 1). The first group, group 1, comprised strains that labeled multiple subgroups categorized as either vibration-sensitive or static deflection-sensitive JO neurons. The second group, group 2, comprised strains that labeled one single subgroup of vibration-sensitive JO neurons.

**Figure 2:**
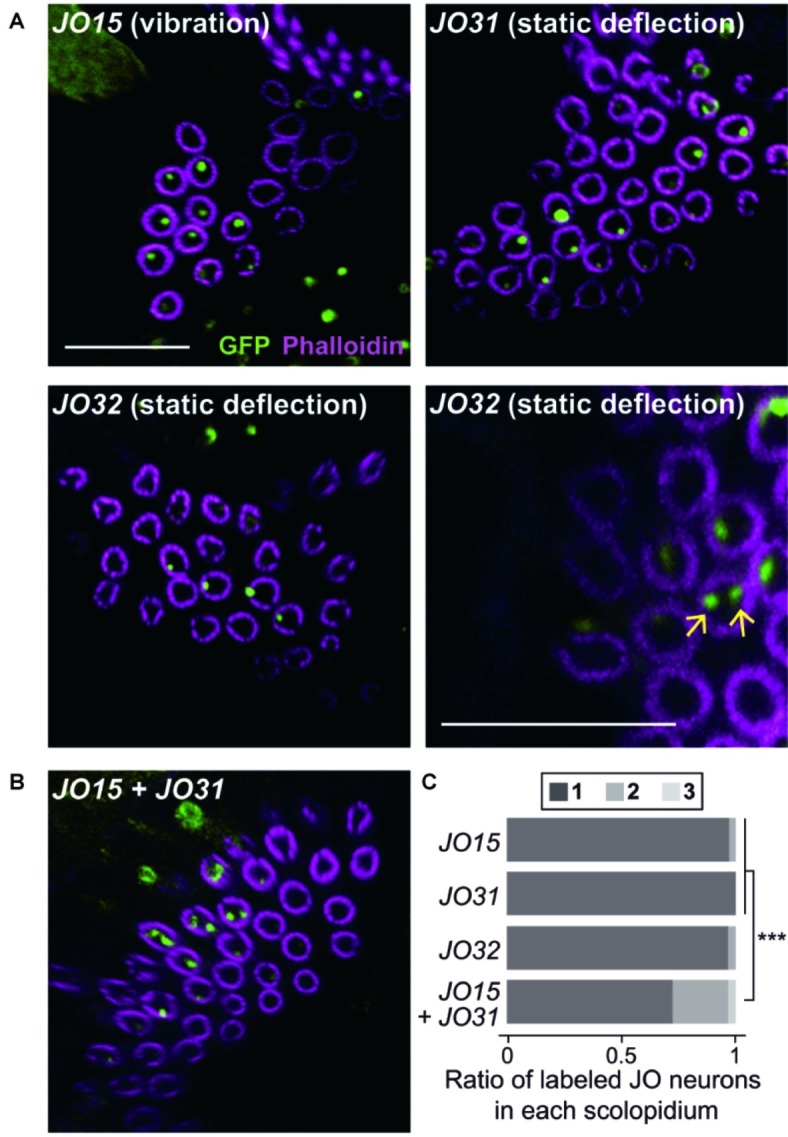
Organization of vibration- and deflection-sensitive JO neurons in scolopidia. Labeled JO neurons in group-1 *GAL4* driver strains that label vibration- or deflection-sensitive subgroups of JO neurons **(A)** and their combination **(B)**. Arrows indicate the examples of two-labeled cilia (green) in a scolopidium (magenta) of *JO32* strain (panel **A**, bottom-right panel). Scale bar = 10 μm. **(C)** The ratio of one, two, and three labeled JO neurons per scolopidium in each *GAL4* driver strain. ***p < 0.001, Fisher’s exact test with the values of *p* adjusted using the Bonferroni method for multiple comparisons.

We first observed group-1 *GAL4* strains to analyze the organization of vibration-sensitive (JO-A and JO-B) and deflection-sensitive (JO-C and JO-E) subgroups in each scolopidium (Figure 2A, Table 1). *JO15*, which labels most (but not all) JO-A and JO-B neurons (Kamikouchi et al., 2006), labeled only one JO neuron in most selected scolopidia (97.4%). Likewise, *JO31* and *JO32*, both of which label most JO-C and JO-E (Kamikouchi et al., 2006), labeled only one JO neuron in most scolopidia (100% in *JO31* and 96.7% in *JO32*; Table 1). These results together suggest that the majority of scolopidia each contain one vibration and one static-deflection JO neuron as a pair.

To test this speculation, we combined two group-1 *GAL4* strains, each of which labels either vibration-sensitive or deflection-sensitive JO neuronal subgroups. When *JO15* and *JO31* were combined, the ratio of single/double/triple-labeled-neuron populations was significantly altered; single-labeled neuron populations decreased from 97.4 and 100 to 72.7% (*p* = 0.00002 for *JO15* vs. *JO15* + *JO31*, *p* = 0.00001 for *JO31* vs. *JO15* + *JO31*, Figures 2B,C; Table 1). This finding suggests that scolopidia that have one vibration-sensitive and one static deflection-sensitive JO neuron as a pair (rather than a pair of vibration sensitive neurons or a pair of static deflection-sensitive neurons) are dominant in the JO.

### Organization of Single Subgroups of Vibration-Sensitive Johnston’s Organ Neurons

**Figure 3:**
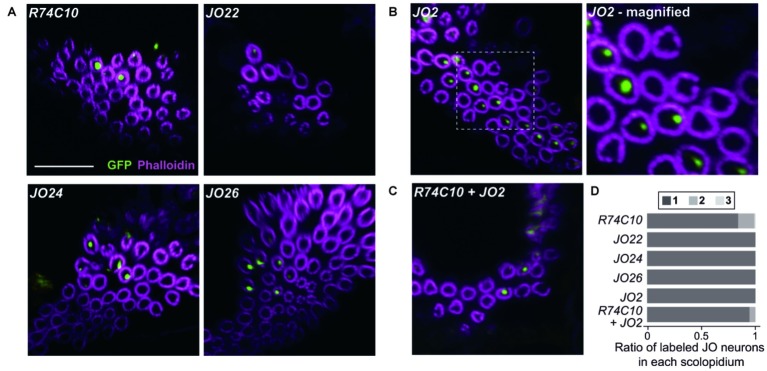
Organization of different subgroups of vibration-sensitive JO neurons in scolopidia. **(A–C)** Labeled JO neurons in group-2 *GAL4* driver strains that label vibration-sensitive JO-A **(A)**, JO-B **(B)**, and their combinations **(C)**. Magnified view of *JO2* is shown. Scale bar = 10 μm. **(D)** The ratio of one, two, and three labeled JO neurons per scolopidium in each *GAL4* driver strain and a combination of two *GAL4* driver strains.

We next observed the organization of single subgroups of vibration-sensitive JO neurons using group-2 *GAL4* strains (Figure 3; Table 1). When we used *R74C10*, which labels most JO-A neurons (Ishikawa et al., 2017), 84.3% of the selected scolopidia housed one labeled JO neuron and 14.8% had two. *JO22*, *JO24*, and *JO26*, which label only a small subset of JO-A (Kamikouchi et al., 2006), labeled only one JO neuron in all the selected scolopidia. This finding suggests that most, but not all, JO-A neurons are not paired with another JO-A neuron in the same scolopidium. When we used *JO2*, which labels most JO-B neurons (Kamikouchi et al., 2006), all the selected scolopidia housed one labeled JO neuron. All JO-B neurons are thus likely to be paired with a JO neuron of another subgroup.

To evaluate whether JO-A and JO-B are housed together in a single scolopidium, we combined two group-2 strains. When we combined *R74C10* and *JO2*, which label most JO-A and JO-B neurons, respectively, the ratio of single/double/triple-labeled-neuron populations was not significantly altered (Figure 3D; Table 1, *p* = 0.066 for *R74C10* vs. *R74C10* + *JO2*, *p* = 0.082 for *JO2* vs. *R74C10* + *JO2*). This observation suggests that most scolopidia have either JO-A or JO-B as a vibration-sensitive JO neuron.

## Discussion

In the present study, we explored the organization of JO neurons in each scolopidium and revealed that most scolopidia had one vibration-sensitive JO neuron and one static deflection-sensitive JO neuron as a pair. This finding suggests that JO neurons are housed in a heterogenous manner – that one JO neuron for hearing and another for sensing gravity and wind are paired within a single scolopidium. JO-B neurons, which selectively respond to low-frequency vibrations, were also singly housed in most scolopidia, suggesting a general rule that multiple neurons with similar response properties are not located in a single scolopidium. A deviation from this rule was observed for JO-A neurons; although most JO-A neurons were housed singly in each scolopidium, some JO-A neurons were paired with another JO-A neuron. A previous report revealed the heterogeneity of JO-A neurons at the anatomic and physiologic levels (Ishikawa et al., 2017), raising the possibility that two heterogeneous JO-A neurons could be housed together in a single scolopidium. Since our analysis was restricted to the scolopedia that are observable from particular orientations, we could not exclude the possibility that these rules might be true only in a specific JO neuron population. Further analyses are required to evaluate this possibility.

Mutual interference may occur between two sensory neurons housed in the same compartment (i.e., scolopidium), as the cilia of the two JO neurons in a scolopidium are in close contact and can influence each other through passive electrical interactions during stimulation. A previous study using an electrical model of a multi-neuron sensillum indicated that gathering identical neuron types in the same sensillum is generally disadvantageous, but may be advantageous if their thresholds differ (Vermeulen and Rospars, 2004). Indeed, insect olfactory sensilla house heterogeneous ORNs (de Bruyne et al., 2001). In *Bombyx mori*, the trichoid sensilla on the antennae house two ORNs, each sensitive to one of the two components of the sexual pheromone, e.g., bombykol or bombykal (Kaissling et al., 1978). In *D. melanogaster*, the ORNs in the olfactory sensilla are clustered in stereotyped combinations and activation of an ORN inhibits its neighboring ORN housed in the same sensillum (de Bruyne et al., 2001; Su et al., 2012). Further studies are needed to evaluate whether such passive (non-synaptic) electrical interactions occur between two JO neurons in a scolopidium.

## Data Availability Statement

The raw data supporting the conclusions of this article will be made available by the corresponding author upon reasonable request.

## Author Contributions

AK and YI contributed to the conception and design of the study and wrote the manuscript. MF, JW, and AU performed the experiments. YI performed the statistical analysis. All authors contributed to manuscript revision, read and approved the submitted version.

### Conflict of Interest

The authors declare that the research was conducted in the absence of any commercial or financial relationships that could be construed as a potential conflict of interest.
